# Silver-Zeolite Combined to Polyphenol-Rich Extracts of *Ascophyllum nodosum*: Potential Active Role in Prevention of Periodontal Diseases

**DOI:** 10.1371/journal.pone.0105475

**Published:** 2014-10-01

**Authors:** Zohreh Tamanai-Shacoori, Fatiha Chandad, Amélie Rébillard, Josiane Cillard, Martine Bonnaure-Mallet

**Affiliations:** 1 Equipe de Microbiologie, EA 1254, Université Rennes 1, UEB, Rennes, France; 2 Groupe de Recherche en Ecologie Buccale, Faculté de médecine dentaire, Université Laval, Québec City, Québec, Canada; 3 Laboratoire Mouvement, Sport, Santé, EA 1274, Université Rennes 1, Université Rennes 2, UEB, Rennes, France; 4 Centre hospitalo-universitaire, Rennes, France; Université de Technologie de Compiègne, France

## Abstract

The purpose of this study was to evaluate various biological effects of silver-zeolite and a polyphenol-rich extract of *A. nodosum* (ASCOP) to prevent and/or treat biofilm-related oral diseases. *Porphyromonas gingivalis* and *Streptococcus gordonii* contribute to the biofilm formation associated with chronic periodontitis. In this study, we evaluated *in vitro* antibacterial and anti-biofilm effects of silver-zeolite (Ag-zeolite) combined to ASCOP on *P. gingivalis* and *S. gordonii* growth and biofilm formation capacity. We also studied the anti-inflammatory and antioxidant capacities of ASCOP in cell culture models. While Ag-zeolite combined with ASCOP was ineffective against the growth of *S. gordonii*, it showed a strong bactericidal effect on *P. gingivalis* growth. Ag-zeolite combined with ASCOP was able to completely inhibit *S. gordonii* monospecies biofilm formation as well as to reduce the formation of a bi-species *S. gordonii*/*P. gingivalis* biofilm. ASCOP alone was ineffective towards the growth and/or biofilm formation of *S. gordonii* and *P. gingivalis* while it significantly reduced the secretion of inflammatory cytokines (TNFα and IL-6) by LPS-stimulated human like-macrophages. It also exhibited antioxidant properties and decreased LPS induced lipid peroxidation in gingival epithelial cells. These findings support promising use of these products in future preventive or therapeutic strategies against periodontal diseases.

## Introduction

Periodontal diseases (PD) are the most prevalent chronic infectious diseases worldwide [1]. These infections lead to progressive destruction of the tooth supporting tissues, bone resorption and tooth loss. Several studies have reported the potential link between PD and systemic diseases including diabetes, and cardiovascular disease. The pathogenesis of PD is associated with accumulation of an oral biofilm (also called dental plaque) and modulation of the host inflammatory response [2]. *Porphyromonas gingivalis,* a Gram-negative anaerobic bacterium [3], [4], is one of the most important pathogens associated with the development of periodontitis. However, *P. gingivalis* requires others bacterial species called early-colonizers including *Streptococcus gordonii* [5], to form the subgingival biofilm. *P. gingivalis* and *S. gordonii* interact with gingival epithelial cells [6], [7] and impair their functions [8]. In addition, *P. gingivalis* secretes a wide range of proteases and degrade various host proteins including collagen, fibronectin, immunoglobulins and cytokines [9]. The host immune reaction to these colonizing bacteria and their products, particularly lipopolysaccharides (LPS), induces the secretion of a wide array of molecules, including cytokines, prostaglandins, proteolytic enzymes and oxidative stress products [1], [10]–[12]. Thus, prevention of PD is focused on the development of strategies to inhibit both bacterial biofilm formation and to modulate the host inflammatory response.

Recently, marine algae have attracted the scientific interest as natural source of bioactive products possessing various biological properties. In fact, several studies have reported that extracts, rich in polysaccharides and polyphenols, from the marine brown alga *Ascophyllum nodosum*, possess various beneficial biological properties including anticoagulant and antithrombotic [13], antitumoral [14], antiviral [15], antioxidant [16], [17] and potent anti-inflammatory effects [18].

On the other hand, ionic silver (Ag^+^) has long been used for its broad-spectrum antimicrobial properties in different fields including dentistry [19], ocular infections and treatment of burns [20], [21]. Advances in chemical technology allowed trapping Ag^+^ within zeolite (a porous crystalline material of hydrated sodium aluminosilicate with a strong affinity for ionic heavy metals). This complex, called silver-zeolite (Ag-zeolite), has been applied to various materials as long-lasting antimicrobial disinfectant [22]–[25].

In the present study, we hypothesized that the biological properties of marine algae extracts combined to those of the complex silver-zeolite could have beneficial effects in the prevention of PD by inhibiting bacterial biofilm formation and modulating the host immune response. Our aims were then to investigate the effects of a polyphenol-rich extract from the algae *Ascophyllum nodosum* called ASCOP (for *Ascophyllum nodosum* polyphenols), combined or not with Ag-zeolite, on bacterial growth and biofilm formation of two oral bacterial species *S. gordonii* and *P. gingivalis*. The anti-inflammatory and antioxidant properties of the ASCOP were assessed in cell culture models.

## Materials and Methods

### Preparation of the complex Ag-zeolite

The complex Ag-zeolite was synthesized by an ion-exchange method [26]. Briefly, constant amounts of zeolite were suspended in 600 mL of demineralised water containing 2.60 g of silver nitrate (AgNO_3_). The solution was stirred for 3 hours at 60°C to obtain a complex formed by zeolite loaded with ionic silver. After a vacuum filtration and three washes in distilled water, the Ag-zeolite complex was dried for 12 hours at 100°C. The obtained suspension was analysed by ICP MS (Inductive Coupled Plasma Mass Spectrometry) to determine the silver proportion in the complex Ag-zeolite. The final solution containing the active complex Ag-zeolite was formed by silver (0.38 µg mL^−1^) and zeolite (0.50 µg mL^−1^) and was distributed into aliquots and stored at −80°C until use in experimental assays.

### Preparation of polyphenol-rich extract of *Ascophyllum nodosum* (ASCOP)

The algae *A. nodosum* was harvested from the west coast of France. A polyphenol-rich extract from *A. nodosum* called ASCOP was prepared following the protocol described by Chandler and Dodds [27]. Briefly, ASCOP was obtained from *A. nodosum* by successive water extraction, alginate precipitation, filtration and final concentration by ultra-filtration procedures. Phenolics concentration was estimated by comparison to a calibration curve prepared with 0–50 µg gallic acid [27]. The final polyphenol-rich solution was lyophilized and stored at 4°C until use. ASCOP mixed with the Ag-zeolite was used in antibacterial and anti-biofilm assays. ASCOP was used alone in antioxidant and anti-inflammatory assays.

### Bacterial culture

*P. gingivalis* ATCC 33277 and *S. gordonii DL1* were grown in brain-heart infusion broth (BHI) (DIFCO, France) and/or on blood Columbia agar plates (AES Chemunex, France) supplemented with hemin (5 g mL^−1^) and menadione (1 g mL^−1^) (Sigma Aldrich, France). *P. gingivalis* cultures were incubated under anaerobic conditions (N_2_-H_2_-CO_2_ [80∶10∶10]) and *S. gordonii* was grown aerobically at 37°C.

### Macrophage-like and gingival epithelial cell cultures

Cells from the human myeloid leukemia cell line U937 (ATCC CRL-1593.2) were grown in RPMI-1640 medium (Sigma-Aldrich). Cells from the human gingival epithelial carcinoma cell line Ca9-22 (Health Science Research Resources Bank, Osaka, Japan) were cultured in Dulbecco's Modified Eagle Medium (DMEM) (Lonza, France) at 37°C in humidified atmosphere of 5% CO_2_. Both cell culture media were supplemented with 10% heat-inactivated fetal bovine serum (FBS, Lonza, France) and antibiotics (100 mg mL^−1^ penicillin/50 mg mL^−1^ streptomycine) (Sigma Aldrich). Monocytic U937 cells were differentiated into macrophage-like cells using 16 nM phorbol-12-myristate-13-acetate (PMA) (Sigma Aldrich) in RPMI-1640 supplemented with 10% FBS for 48 hours. The adherent macrophage-like cells were washed and incubated for an additional 24 hours in fresh RPMI-1640-10% FBS medium. For all the experiments, a cellular count was carried out using a haemocytometer and the cells were suspended in RPMI supplemented with 1% FBS, seeded in six-well plates (1×10^6^ cells/well/1 mL) and incubated at 37°C in a 5% CO2 atmosphere for 2 h prior to the ASCOP treatment and lipopolysaccharide (LPS) stimulations.

### Antibacterial assay

The capacity of ASCOP mixed with Ag-zeolite to inhibit bacterial growth was assessed using a standard procedure according to the European norms prEN 1040 (AFNOR: NF EN 1040-1997) in chemical disinfectants and antiseptics section. Briefly, 1–5×10^7^ cfu mL^−1^ of a fresh bacterial suspension (standard inoculum) was incubated in the presence of the tested product for 15 min at 15°C. For controls, the same experiments were carried out in the same conditions with sterile BHI but without the tested product. For bacterial viable count, samples (100 µL) of serial dilutions were plated onto blood Columbia agar plates and incubated at 37°C for 48 hours in aerobiosis for *S. gordonii*, and for 6 days, under anaerobic conditions for *P. gingivalis*. Each experiment was repeated three times and each colony count was repeated two times. The results were expressed as log_10_ values of the mean colony forming unit (cfu) count.

### Anti-biofilm formation assay

The effect of the tested product on biofilm formation has been assessed using the Biofilm Ring Test method (BioFilm Control, Saint Beauzire, France) as described by the manufacturer. This assay is based on the immobilization of magnetic beads when they become embedded in bacterial aggregates. Briefly, bacterial inoculums (mono-specie and/or bi-species) of *P. gingivalis* and/or *S. gordonii* were prepared in BHI medium and adjusted to 10^7^–10^8^ cfu mL^−1^. Bacteria (200 µl) were distributed in wells of polystyrene 96-well plates and mixed with 10 µL mL^−1^ of the toner solution (TON 005 containing magnetic beads, provided by the manufacturer) in the presence or absence of the test product. Controls without bacteria were also run in the assay to be sure that the tested product does not interfere with the beads. After 3 hours incubation at 37°C in an anaerobic atmosphere, plates were scanned before and after magnetization using the dedicated Scan Plate Reader (BioFilm Control, France) operated with the Biofilm Control Software (BioFilm Control, France). The biofilm formation was expressed as a Biofilm Formation Index (BFI) calculated by the software and based on attracted beads forming a black spot in the bottom of the wells and detected by the Scan Plate Reader. Calculated BFIs are inversely proportional to the biofilm formation density. A BFI value ≤2 corresponds to a densely formed biofilm whereas a BFI value ≥12 indicates the absence of biofilm formation. The results are expressed as the mean of BFI values of triplicate from three independent experiments.

### Cytotoxicity assay

Eukaryotic cell viability was measured by MTT assay [3-(4, 5-dimethylthiazol-2-yl)-2, 5-diphenyl tetrazolium bromide (MTT; Sigma-Aldrich)]. The U937 macrophage-like cells (5×10^3^ cells/well), distributed in wells of 96-well plates, were exposed to increasing concentrations of ASCOP solution (0.10, 0.25 and 0.50 µg mL^−1^ in RPMI-1640 culture medium) with three wells for each concentration. After 24 hours incubation in the presence or absence of a bacterial stimuli (0.5 µg mL^−1^ *P. gingivalis* LPS (invivoGen, Toulouse, France), 10 µL of MTT (0.50 mg mL^−1^ final concentration) were added to each well. After 4 hours incubation at 37°C, the supernatant was removed and 100 µl of acidic (0.04 M HC) isopropanol were added to each well to solubilize the formazan crystals. After vigorous shaking, absorbance was measured using a microplate reader at 550 nm wavelength. A correction was carried out at 655 nm to exclude the cellular debris. The cellular percentage of viability was expressed according to the following formula: OD (optical density) of cells treated with ASCOP solution x 100/OD of untreated cells. The ASCOP concentrations showing less than 20% of cell mortality were selected as working concentrations for the subsequent experiments.

### Anti-inflammatory activity

Macrophage-like cells U937 (5×10^6^ cells/wells of six-well plates) were pre-incubated for 2 hours with non-toxic concentrations of ASCOP (0.10 µg mL^−1^), and then stimulated for an additional 24 hours period with 0.5 µg mL^−1^ of *P. gingivalis* LPS. After the incubation period, the amounts of inflammatory cytokines TNFα and IL-6 were measured in the cell culture supernatants using commercial ELISA kits (R&D Systems, Minneapolis, MN) according to the manufacturer's protocol. Non LPS-stimulated and non ASCOP treated cells were used as negative controls. Cells stimulated with LPS but not treated with ASCOP also were used as positive controls of stimulation. Three independent experiments were conducted in triplicate and data are expressed as means ± SD.

### Antioxidant activity of ASCOP

#### Free Radical scavenging activity

Superoxide anion (O2^−^) was produced by the xanthine/xanthine oxidase system and hydroxyl radical (OH) was generated from decomposition of H_2_O_2_ by ferrous ions. Both reactions were carried out in the presence of a spin trap 5,5-dimethylpyrroline-N-oxide (DMPO). ASCOP solution was added at concentrations ranging respectively from 0.025 to 0.50 µg mL^−1^ for superoxide anion assay and 0.0025 to 0.05 µg mL^−1^ for hydroxyl radical assay. Electron Spin Resonance (ESR) spectra were recorded at room temperature using a Bruker ECS 106 spectrometer. The scavenging activity of ASCOP was evaluated by the decrease in the ESR signal expressed as arbitrary units. Scavenging activities were calibrated using the bovine erythrocyte superoxide dismutase (SOD, Sigma-Aldrich) as standard for superoxide anion, and benzoic acid as reference scavenging substance for hydroxyl radical. All ESR experiments were performed five times for each point and data are expressed as means ± SD.

#### Total Antioxidant Capacity

The Oxygen Radical Absorbance Capacity (ORAC) experiments were carried out according to Prior *et al.* [28]. ASCOP was used at a concentration of 0.10 µg mL^−1^. A standard curve was performed using Trolox (6-hydroxy-2,5,7,8-tetramethylchroman-2-carboxylic acid) (Sigma Aldrich), a synthetic analogue of vitamin E, at concentrations ranging from 6.20 µM to 50 µM. Final values were expressed as micromole Trolox equivalents per gram. Trolox experiments were performed five times and the results are expressed as the mean ± SD of spectrophotometric absorbance.

### Anti-lipoperoxidation activity

Ca9-22 gingival epithelial cells were seeded at 1×10^5^ cells/well of 96-well plates in DMEM-10% FBS medium. After 24 hours incubation, cells were treated with ASCOP solution (10 µg mL^−1^) for 1 hour before being stimulated with *P. gingivalis* LPS of (10 µg mL^−1^) during 2 hours. Lipid peroxidation as a marker of oxidative stress was evaluated by measurement of 15 F_2_ α-Isoprostanes (also called 8-Isoprostanes) released in the culture medium. F2 α-Isoprostanes were analyzed by LC-MS according to the protocol previously described [29]. Each experiment was performed in triplicate and repeated three times. Concentrations of isoprostanes (picogramme isoprostanes/mL) are expressed as means ± SD.

### Statistical analysis

All data are expressed as means ± standard deviations of triplicates from at least three independent experiments. The significance of the results was assessed using the Student's *t*-test. An effect was considered statistically significant if its *p* value was lower than 0·05 (*p*<0.05 as significant) except in the case of antioxidant assay, it was lower than 0.01 (*p*<0.01 as significant).

## Results

### Antibacterial activities

After 60 min of exposure, the antibacterial activity of the tested products against *S. gordonii* and *P. gingivalis* were evaluated according to the European norms (Table 1). Ag-zeolite in the presence or absence of ASCOP showed strong bactericidal effect (*p*<0.05) by complete killing of *P. gingivalis*. In the same condition, silver alone at 0.38 µg mL^−1^ concentration revealed a weaker effect on the growth of *P. gingivalis* with 3 log reduction (*p*<0.05) (Table 1) compared to the starting inoculum. Both zeolite (0.50 µg mL^−1^) and ASCOP (0.50 µg mL^−1^), independently assessed, did not show significant effect on the growth of *P. gingivalis*.

**Table 1:** Bacterial count (cfu mL^−1^) of *S. gordonii* and *P. gingivalis* cultures after 60 min treatment with silver (Ag), ASCOP, zeolite, Ag-zeolite or Ag-zeolite+ASCOP.

*Bacterium*	*Control*	*Ag*	*ASCOP*	*Zeolite*	*Ag-zeolite*	*Ag-zeolite+ASCOP*
***S. gordonii*** DL1	2,16×10^7^±0,07	2,99×10^7^±0,12	2,91×10^7^±0,15	2,17×10^7^±0,17	2,70×10^7^±0,23	2,56×10^7^±0,25
***P. gingivalis*** ATCC33277	2,33×10^7^±0,29	2,59×10^4^*±0,40	2,90×10^7^±0,45	1,23×10^7^±0,25	0*	0*

Control: in absence of active products.

The values are means ±standard deviations for measurements obtained for three independent experiments (* *p <0.05; n = 6*).

For *S. gordonii*, none of the evaluated products showed any effect on the growth of this bacterial species. The number of bacteria (cfu mL^−1^) remained similar to that of the non-treated control after 60 min of treatment with the tested products.

### Anti-biofilm formation properties

**Figure 1:**
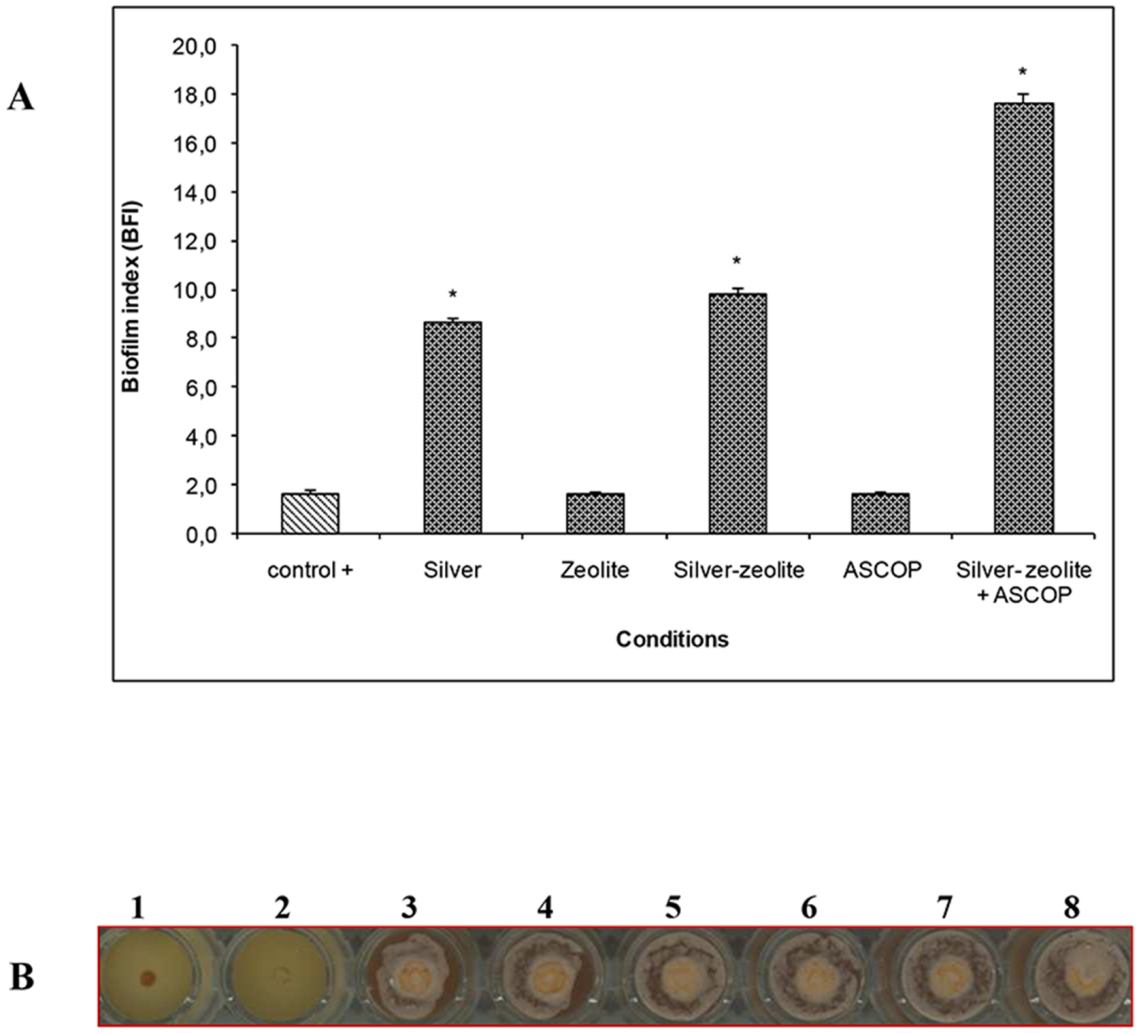
Biofilm formation of monoculture of *S. gordonii* treated with silver (Ag), ASCOP, zeolite, Ag-zeolite or Ag-zeolite+ASCOP. **A**: BFI (biofilm formation index) values for monoculture of *S. gordonii* in absence or presence of silver (Ag); zeolite; Ag-zeolite; ASCOP or Ag-zeolite+ASCOP. *Significantly different from control (*p*<0.05; n = 9). **B**: Biofilm formation of *S. gordonii* in the presence of Ag-zeolite combined to ASCOP. Well 1 (BHI, negative control), well 2 (positive control), wells 3 to 8 (replicate of *S. gordonii* in the presence of Ag-zeolite+ASCOP.

**Figure 2:**
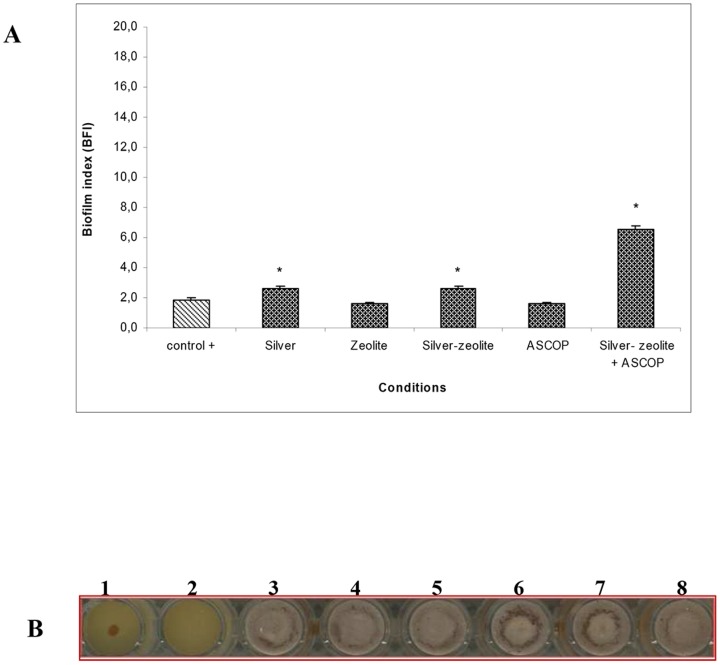
Biofilm formation of the coculture of *S. gordonii + P. gingivalis* treated with silver (Ag), zeolite, Ag-zeolite, ASCOP or Ag-zeolite+ASCOP. **A**: Biofilm formation of the coculture of *S. gordonii + P. gingivalis* treated with silver (Ag), zeolite, Ag-zeolite, ASCOP or Ag-zeolite+ASCOP. *Significantly different from control (*p*<0.05; n = 9). **B**: Biofilm formation of the coculture *S. gordonii + P. gingivalis* in the presence of Ag-zeolite combined to ASCOP. Well 1 (BHI, negative control), well 2 (positive control), wells 3 to 8 (replicate of the coculture *S. gordonii + P. gingivalis* in the presence of Ag-zeolite+ASCOP.

The capacity of Ag-zeolite, combined or not to ASCOP, to inhibit biofilm formation by *S. gordonii in* monoculture or in coculture with *P. gingivalis*, was investigated. The BFI for *S. gordonii* monoculture biofilm or in combination with *P. gingivalis* (positive controls) were respectively 1.65±0.15 and 1.85±0.15. However, when bacteria were treated with Ag-zeolite combined to ASCOP, biofilm forming capacity of *S. gordonii* in monoculture or coculture with *P. gingivalis* was altered (Figures 1 and 2). Indeed, *S. gordonii* biofilm formation was completely inhibited in the presence of the complex Ag-zeolite combined to ASCOP (Figures 1A and 1B). The BFI reached 17.50±0.20 (*p*<0.05) in the case of *S. gordonii* monoculture biofilm and 6.42±0.10 (*p*<0.05) for the coculture *S. gordonii* + *P. gingivalis* biofilms (Figures 1A and 2A). The capacity of *S. gordonii* to form a biofilm was less affected by Ag-zeolite in the absence of ASCOP (BFI of 9.95±0.15) (*p*<0.05) (Figure 1A). Under the same conditions, a slight effect was observed on the biofilm formation of the coculture *S. gordonii + P. gingivalis* (BFI = 2.6±0.11) (*p*<0.05) (Figure 2A).

### Anti-inflammatory activities of ASCOP

**Figure 3:**
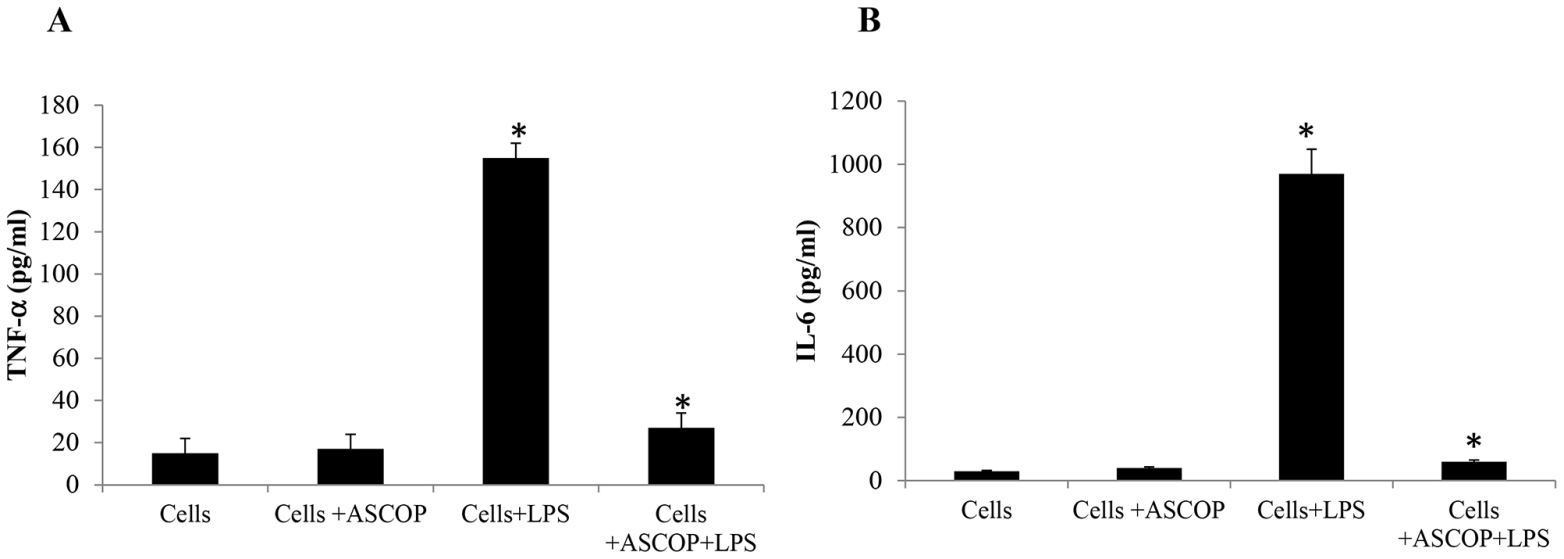
Cytokine measurement in cell culture medium of macrophage like cells U937. Levels of TNFα (**A**) and IL-6 (**B**) in culture supernatant of macrophage-like cells U937 stimulated with LPS (0.5 µg mL^−1^) and treated or not with ASCOP (0.1 µg mL^−1^). Values are expressed as means ± standard deviation of triplicates (* *p*<0.05).

To investigate the effects of ASCOP on inflammatory cytokine (TNFα and IL-6) production, macrophage-like cells U937 were treated with ASCOP (0.10 µg mL^−1^ solution) before stimulation with LPS. Cytokine productions were measured in the cell culture medium by ELISA. As shown in Figure 3, ASCOP treatment caused significant decrease (*p*<0.05) of the secretion of TNFα (Figure 3A) and IL-6 (Figure 3B) in LPS stimulated macrophage-like cells. The amounts of TNFα and IL-6 in the supernatant of treated cells were clearly less than those of the LPS stimulated control cells (94% and 84% of reduction respectively). ASCOP had no effects on unstimulated control cells (Figures 3A and B). To exclude the possibility that the decrease in cytokine amounts might have been caused by cytotoxic effects of ASCOP solution, we have evaluated the viability of the macrophage-like cells by an MTT assay. No cytotoxic effects were detected following treatments of macrophages with increasing concentrations of ASCOP ranging from 0.10 to 0.50 µg mL^−1^, and cell viability was ≥98% of the untreated controls in all experiments (data not shown).

### Antioxidant activities of ASCOP

ASCOP at a concentration of 0.50 µg mL^−1^ decreased DMPO-OOH signal by 50%. The superoxide anion scavenging capacity of ASCOP was calculated to be equivalent to 207 UI SOD [30]. For hydroxyl radical, ASCOP at a concentration of 0.05 µg mL^−1^ decreased DMPO-OH signal by 50%. Hydroxyl radical scavenging capacity of ASCOP was determined to be equivalent to 83 mg mL^−1^ of benzoic acid. It should be mentioned that in these experiments, the concentrations of ASCOP used to scavenge hydroxyl radical were 10 times lower than those used to scavenge superoxide anion. This is due to a higher reactivity of hydroxyl radical compared to superoxide anion.

The total antioxidant capacity (TAC) of ASCOP determined by ORAC assay was calculated to be 296 micromole Trolox equivalents per gram.

**Figure 4:**
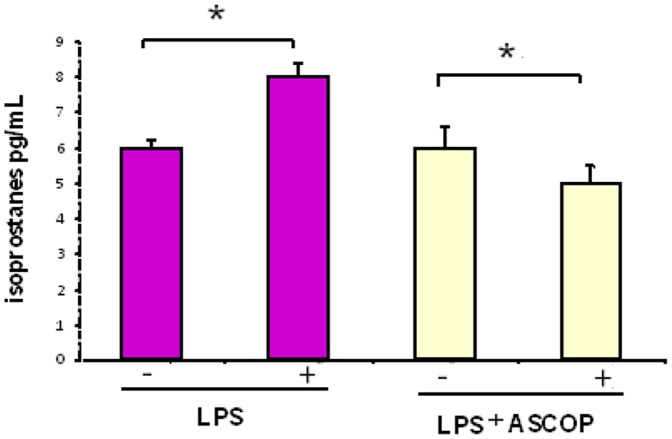
Isoprostanes release in culture media of epithelial gingival cells Ca9-22. Cells were treated with ASCOP (0.10 µg mL^−1^) for 1 hour before been stimulated with LPS (10 µg mL^−1^) during 2 hours. Data were expressed as means of pg isoprostanes per mL of culture medium for a minimum of three independent experiments (*: *p*<0.01).

An inhibition of lipid peroxidation was observed in Ca9-22 epithelial cells treated with 0.10 µg mL^−1^ of ASCOP during 1 h before stimulation with *P. gingivalis* LPS as showed by the significant decrease of isoprostanes in culture medium (*p*<0.01) (Figure 4). No obvious effect of LPS (10 µg mL^−1^) on the viability rate of Ca9-22 cells was observed after 4 h and 24 h of cell stimulation (data not shown).

## Discussion

Over the past two decades, natural compounds with antibacterial, anti-inflammatory and/or anti-oxidant properties have received considerable attention as new therapeutic agents for the prevention and treatment of various infections. In this work, we investigated the biological properties of a polyphenol-rich extract from the marine algae *A. nodosum*, named ASCOP, combined with a complex of silver-zeolite named Ag-zeolite. To the best of our knowledge, no study has described before the bactericidal effect of algae extracts on oral pathogens. Nevertheless, bacteriostatical activities against positive and negative Gram bacteria have already been described for tannins extracted from the algae *Ecklonia kurome*, *E. cava* and *Fucus vesiculosus*, suggesting their potential use as natural preservatives in food industry or as antibacterial drugs [31], [32]. In addition, algae extracts like phytochemical extracts have an advantage over synthetic antibacterial agents in that, pathogens are not frequently exposed to these natural compounds and should not develop resistance. On the other hand, the metallic silver, loaded onto several inorganic systems such as zeolites, was reported as efficient anti-microbial agents [33], [34]. Indeed, the incorporation of silver into zeolite increases ions interaction with microorganism via a higher specific surface area. Lima *et al.* [35] reported that gold nanoparticles dispersed on zeolites are excellent biocide to rapidly eliminate *Escherichia coli* and *Salmonella typhi*. In our study, Ag-zeolite alone or in combination with ASCOP exhibited a high bactericidal activity against *P. gingivalis* while the viability of *S. gordonii* was not affected. This feature could be attributed to the nature of bacterial membrane of the Gram positive bacterium which seems to be more effective in the protection against any cytotoxic effects of these compounds. For ASCOP preparation, no antibacterial effect against *P. gingivalis* or *S.gordonii* was observed.

In addition, we show in this study, for the first time, that Ag-zeolite combined with ASCOP exhibits a marked synergistic effect in alteration of the biofilm forming capacity of *S. gordonii* alone or in coculture with *P. gingivalis*. This result becomes highly pertinent when we consider that the adherence of *P. gingivalis*, one of the late colonizers in the biofilm, depends on its capacity to adhere to the first colonizers including *Streptococcus spp*. [36]. Without affecting *S. gordonii* viability, addition of ASCOP to Ag-zeolite inhibited coculture *S. gordonii* + *P. gingivalis* biofilm formation while silver-zeolite alone did not reveal any specific anti-biofilm effect. This inhibitory effect is probably associated with a different interaction between ASCOP and Ag-zeolite conferring thus a new biological property.

Actually, there is no doubt that plaque bacteria are necessary to initiate the chronic inflammatory response in the periodontal tissues [37]. At the same time, there is strong evidence that destructive processes occurring as part of the host inflammatory response are responsible for the majority of soft-tissue breakdown leading to periodontal diseases. Chronic periodontitis occurs mainly as a result of activation of host-derived immune and inflammatory mechanisms. Cytokines are inflammatory mediators that play a major role in the pathogenesis of periodontal disease and tissue destruction [38], [39]. There is a need to develop new and effective preventive and treatment approaches for periodontal diseases, based on recent advances in host modulation and inflammation resolution [40]. In the present study, we showed that ASCOP significantly decreases the production of two cytokines TNFα and IL-6 in a cell culture model of LPS stimulated macrophages. These results are in agreement with those of others. Studies of Dutot *et al*. [41] reported that a phlorotannin-rich natural extract from *A. nodosum* inhibits the release of pro-inflammatory cytokines. Another study from Bahar *et al*. [42] have also reported that extracts from *A. nodosum* seaweed have potential to suppress the pro-inflammatory response induced by the bacterial LPS in a pig colon model by suppressing LPS-induced pro-inflammatory cytokines IL-8, IL-6, and TNFα genes. Since cytokine release is associated with inflammation and destruction tooth-supporting tissue, these data suggest that ASCOP may contribute to reduction of host cell damage by decreasing secretion of inflammatory mediators [43], [44]. Of note, the results in this study are obtained in a macrophage-like cell culture model using an established cell line. Further studies using primary human gingival cells should be performed to confirm this anti-inflammatory properties.

Our results also revealed that ASCOP exhibits antioxidant properties. Indeed ASCOP revealed a capacity to scavenge free radicals such as superoxide anion, hydroxyl radical and peroxyl radical. Free Radical scavenging activity of *A. nodosum* extracts have been previously reported by others [41]. O'Sullivan *et al*. [45] reported that among five different seaweed extracts, *Ascophyllum* showed the highest capacity to scavenge the free radical DPPH (2,2-diphenyl-1-picrylhydrazyl). Wang *et al*. [46] also showed a high correlation between total phenol content of *A. nodosum* and its high scavenging activity towards DPPH and peroxyl radical (ORAC). Recently, Abu *et al*. [17] showed that ascophyllan, a sulfated polysaccharide from *A. nodosum*, was more potent scavenger of superoxide anion than fucoidan. Nonetheless, ascophyllan and fucoidan showed similar scavenging activity towards hydroxyl radical.

In the present study, ASCOP inhibited oxidative stress by reducing lipid peroxidation induced by *P. gingivalis* LPS in gingival epithelial cells. In fact, bacteria produce a number of metabolites, which affect gingival tissue. One irritating agent, LPS is a major constituent of the outer membrane of Gram-negative bacteria and a critical determinant in initiation and progression of periodontal disease. It has been observed that LPS induces the production of reactive oxygen species (ROS) that could initiate lipid peroxidation [47]. Recent studies have demonstrated that epithelial cells also have a functionally active NADPH-oxidase complex for ROS production and oxidative stress responses [48]. Thus in our model, lipid peroxidation observed in LPS-stimulated epithelial gingival cells could involve the activation of NADPH oxidase by LPS leading to superoxide anion production and as a consequence to hydroxyl production. This later free radical is well known to oxidize polyunsaturated fatty acids in membranes. It has already been reported that application of the polyphenolic fraction of *Lonicera caerulea* fruit to LPS-treated human gingival fibroblasts significantly decreased ROS production, a marker of lipid peroxidation [49].

In summary, this study showed that ASCOP added to Ag-zeolite confers synergistic antibacterial and anti-biofilm properties to this complex and that ASCOP alone possess anti-oxidative and anti-inflammatory activities. Ag-zeolite and ASCOP act on oral bacteria and the host immune response, two etiological components involved in periodontitis. Their combination could be a promising compound in prevention and treatment of periodontal diseases. However, the perspectives of development of potential preventive or therapeutic product, containing ASCOP combined with Ag-zeolite, should be validated in *in vivo* models and should be more optimized in terms of galenic formulation and optimal concentrations presenting the minimal side effects on buccal cells and normal flora.
